# Boosting nutritional value: the role of iron fortification in meat and meat products

**DOI:** 10.1007/s10534-024-00659-1

**Published:** 2025-01-21

**Authors:** Ahmed Hamad, Pallavi Singh

**Affiliations:** 1 Department of Food Hygiene and Control, Faculty of Veterinary Medicine, Benha University, Banha, 13736 Egypt; 2 Faculty of Public Health, Poornima University, Jaipur, Rajasthan India

**Keywords:** Fortification, Food system, Biofortification, Nutritional diseases, Nutritional enhancement

## Abstract

Iron deficiency is a widespread nutritional problem affecting millions of people globally, leading to various health issues including anemia. Iron fortification of meat and meat products has emerged as an effective strategy to combat this issue. This review explores the process and benefits of iron fortification, focusing on the types of iron compounds suitable for fortification, such as ferrous sulfate and ferric pyrophosphate, their bioavailability, and their impact on the sensory and nutritional qualities of meat products. Technological challenges and solutions, including encapsulation, chelation, and microencapsulation techniques, have been examined to minimize their negative impacts on sensory qualities. This review also discusses the regulatory framework governing iron fortification and consumer acceptance. Analytical methods for determining iron content, such as spectrophotometric and colorimetric detection, are discussed. Although iron-fortified meat products offer health benefits, sensory aspects and consumer acceptance are important considerations. This review provides a comprehensive understanding of the role and significance of iron fortification in meat products as a public health intervention to address iron deficiency.

## Introduction

Dietary iron (Fe) deficiency is the most common nutritional disorder, affecting approximately 2 billion people worldwide. Red meat is considered a good source of bioavailable Fe; however, its consumption is not sufficient to solve the Fe-deficiency problem in the poorest populations (Shubham et al. 2020; Natekar et al. 2022). Inadequate iron (Fe) intake is a nutritional problem that affects a large part of the global population and is one of the most prevalent nutritional problems. Its worldwide prevalence is estimated to be 25%. It is present in all social groups, and exists in both developing and developed countries (Al-Naseem et al. 2021; Kumari and Chauhan 2022).

**Fig. 1:**
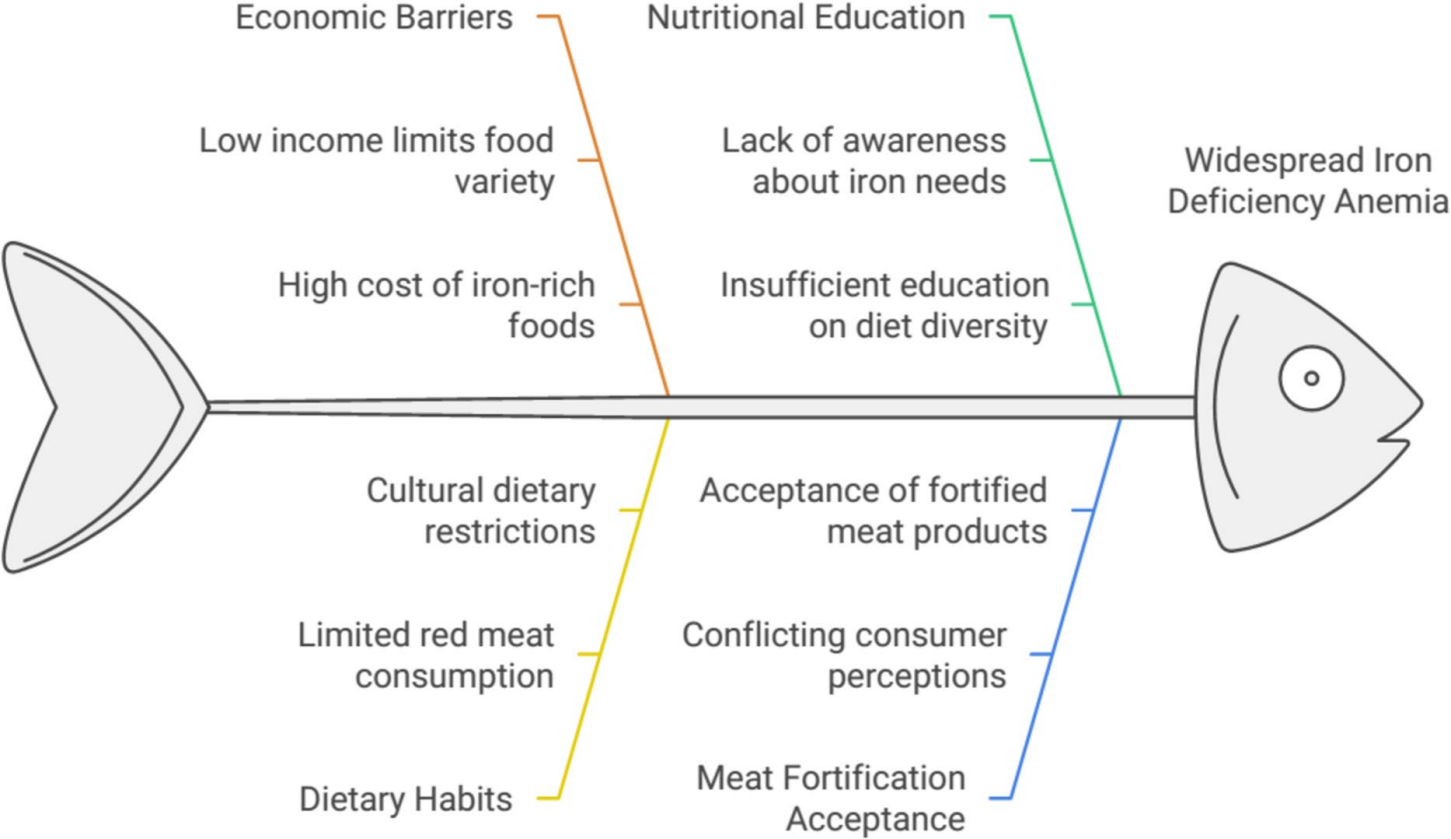
Addressing iron deficiency anemia through meat fortification

Iron deficiency is a major public health problem, mainly affecting infants, pregnant women, and fertile women, especially those from low-income classes. Anemia as a result of Fe deficiency is a common manifestation, constituting the most common cause of anemia and the most frequent nutritional problem in public health, and is present in more than 2 billion people (more than 30% of the world population). As a rule, these populations do not have enough money to buy a sufficient quantity and variety of food for a healthy diet. Supplementation of the diet with low-cost natural food products produced from local raw materials is considered a sustainable and effective method to correct Fe-deficiency anemia in these populations. Most of the world's production of pork, chicken, and turkey is destined for the human diet and, therefore, could be a successful vehicle to resolve these problems using meat products (Cappellini et al. 2019; Rusu et al. 2020; Benson et al. 2021; Pasricha et al. 2021) (Fig. 1).

There are conflicting data on the impact of the addition of Fe to meat on growing consumer acceptance. Therefore, this review provides information on designing meat solutions to prevent Fe deficiency anemia and presents the results of a new techniques used for meat fortification with iron.

## Importance of iron in human nutrition

Iron plays a vital role in numerous metabolic, growth, and developmental processes in the human body, including erythropoiesis, DNA synthesis, and electron transport (Dixit et al. 2021). It is essential for oxygen transport, immunity, cell division, and energy metabolism (Piskin et al. 2022). The average daily dietary iron intake in humans is 10 to 15 mg, with only 1 to 2 mg absorbed through the intestinal system (Piskin et al. 2022).

Interestingly, iron balance in the human body is regulated solely by absorption, as there is no physiological mechanism for excretion (Hurrell and Egli 2010). This unique characteristic sets iron apart from other minerals and underscores the importance of proper dietary intake and absorption. Additionally, the interaction between iron and the gut microbiome has been observed, with iron deficiency potentially altering microbiome structure and promoting the growth of pathogenic bacteria (Sun et al. 2024).

Several authors have attempted to determine whether iron from meat is superior to other foods. It has been shown that increasing both the level and the frequency of meat intake improves iron status, increasing serum ferritin levels independently of the level of dietary enhancers of iron absorption by following meat intake (Sun and Weaver 2021; Rahfiludin et al. 2021). Furthermore, it has been verified that the hemoglobin levels and rates of iron depletion and deficiency anemia in individuals who consume fresh foods are consistently higher than those in individuals who do not. The early onset of iron deficiency in the first months after birth suggests that body reserves of iron do not last long and that the iron sources consumed before the recommended beginning of complementary feeding are important for the prevention or postponement of anemia (Chouraqui 2022; Skolmowska and Głąbska 2022).

**Fig. 2:**
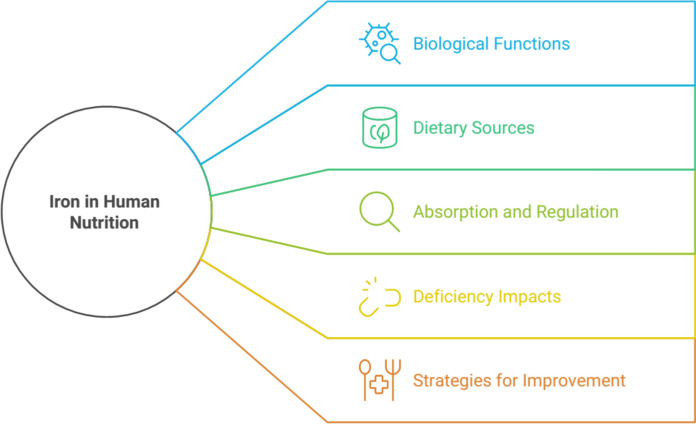
Exploring the multifaceted role of iron

Iron is an essential nutrient that is required for various critical biological functions in the human body. Iron absorption is especially important in the first months of life, when the body is expanding rapidly, and iron reserves from the mother are decreasing. Post-weaning children can suffer from anemia if an appropriate complementary food rich in bioavailable iron is not introduced to their diet. Men and women, elderly people, and pregnant women are also at a high risk of iron deficiency if iron intake is not sufficient to meet the physiological loss and different requirements (Shubham et al. 2020; Juárez et al. 2021; Xing et al. 2022). It is noteworthy to highlight that meat and conserved meats are the food products enabling the highest absorption of iron (Ursachi et al. 2020; Ulloa-Saavedra et al. 2022) (Fig. 2).

Maintaining adequate iron levels is crucial for human health, particularly during periods of rapid growth such as adolescence (Mesías et al. 2013). Iron deficiency can lead to anemia and adversely affect cognitive ability and behavior (Mesías et al. 2013). To combat iron deficiency, various strategies have been proposed, including improving dietary diversity, food iron fortification, increasing intake of iron nutritional supplements, and iron biofortification (Luo et al. 2018). However, the bioavailability of iron from different dietary sources and the influence of various factors on its absorption remain important considerations in addressing iron deficiency globally (Hunt 2005; Hurrell and Egli 2010).

## Iron deficiency and anemia

Iron in the human body is mostly present in the erythrocytes. Iron controls the richness and maturation of erythroid precursors, in relation to the need for oxygen in the human body. When iron transport is insufficient to meet oxygen demand, erythrocyte production decreases. Iron is also a constituent of myoglobin in muscle tissues; it plays a vital role in the mitochondrial electron transport chain and cellular metabolism and is essential for the biosynthesis of purine nucleotides, deoxyribonucleotides, and sulfur-containing enzymes (Wilson and Reeder 2022; Kang et al. 2023). Iron is present in many other enzyme reactions that control the metabolism of organisms, including the oxidation of fatty acids. In circumstances of iron deficiency, the synthesis of these enzymes is impaired, regardless of whether they are heme-containing or heme-independent. As with many nutrients in the body, iron levels need to be regulated because dietary iron can accumulate beyond toxic levels (Alves et al. 2021; Vinke et al. 2023).

**Fig. 3:**
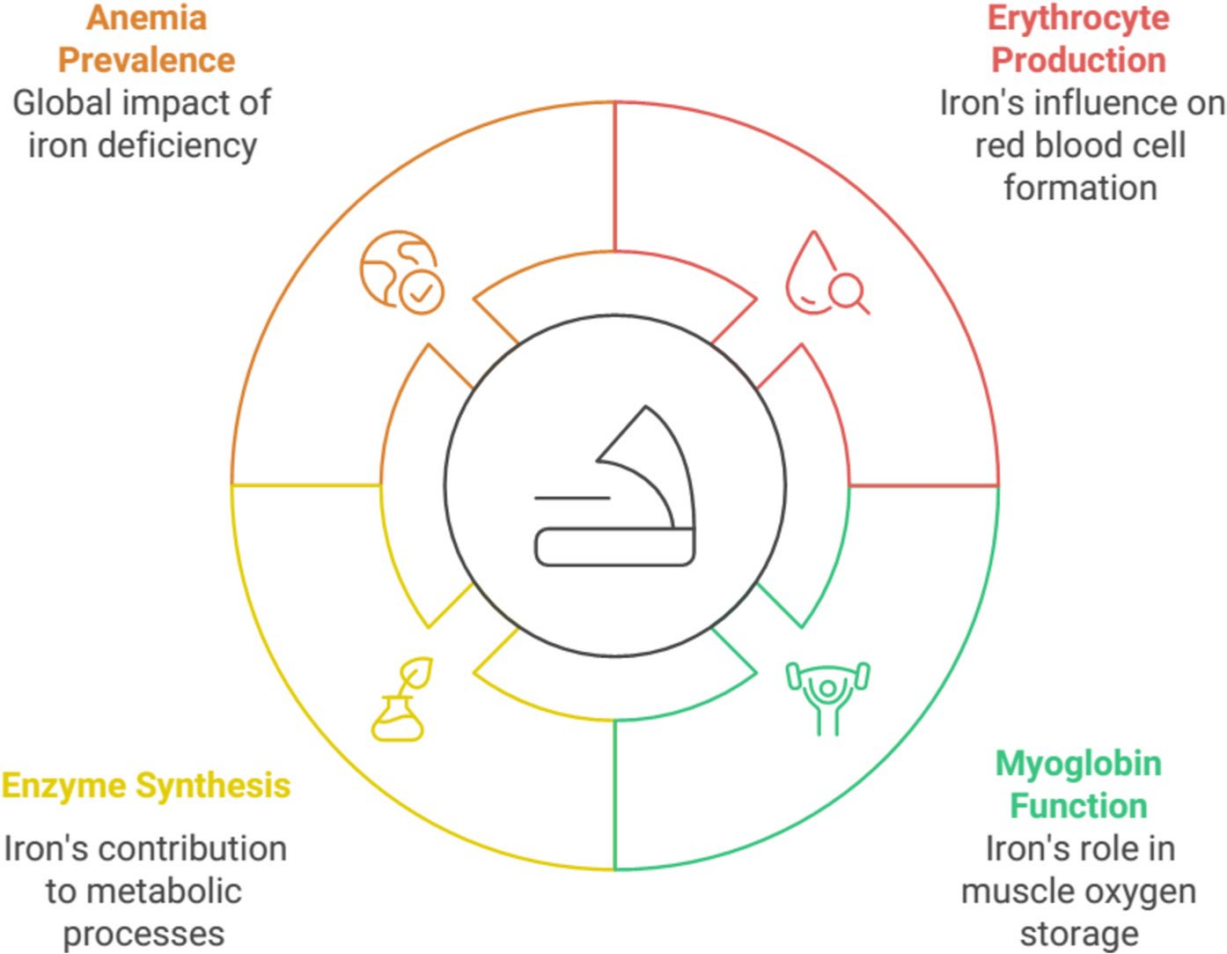
Iron's role and anemia outcomes

Anemia is a global public health problem affecting both developing and developed countries. Although the prevalence of anemia has declined over the last few decades, anemia control programs have only been implemented in developing countries. The World Health Organization (WHO) defines anemia as a state in which the hemoglobin (Hb) level is decreased because of insufficient oxygen-carrying capacity of erythrocytes. Although anemia has many potential causes, lack of iron is a major cause of anemia. In adults, anemia affects half of the pregnant women, 30% of the elderly, and 40% of non-pregnant women, whereas only 20% of the cases are due to iron deficiency (Cappellini et al. 2019; Romano et al. 2020; Snook et al. 2021) (Fig. 3).

## Iron fortification: definition and purpose

Iron fortification is a widely used strategy to address iron deficiency, which affects approximately 30% of the world's population (Perera et al. 2023). Compensating iron deficiency by fortifying foods with low energy, energy-dense, accessible, inexpensive, and at the same time not causing serious sensory and technological disadvantages is a desirable goal. It has good bioavailability and is a nutrient that is scarce, especially in poor societies and countries (Mayer Labba et al. 2022; McClements and McClements 2023). The addition of a given nutrient (in this case iron) is called fortification. Traditional methods for food fortification include the addition of iodine to salt or folic acid to flour, biotechnological methods, microencapsulation methods, and microencapsulated iron. In South Sudan, red speckled beans are generally released with 80% higher iron content and can be exposed to different fortification methods (Shubham et al. 2020; Hurrell 2021).

## Techniques of iron fortification in meat products

Meat and meat products are excellent carriers of haem iron; however, meat-rich diets may lead to other health problems. It is important to ensure that the population will continue to consume a diet that provides an adequate intake of highly bioavailable, as well as poorly bioavailable, but not excessive iron (Macho-González et al. 2021; Xing et al. 2022).

The latter can be provided by non-heme iron, which is a standard fortification technique for many other processed foods. The main objective of iron fortification in meat products is to improve micronutrient intake by consumers without significantly changing their eating habits. The development of iron-fortified functional meat products is an innovative strategy for overcoming iron deficiency in populations without changing their eating habits. In this context, the topic of iron fortification of meat and meat products is discussed. The use of iron fortificants such as ferrous sulfate or ferric pyrophosphate is common in the meat industry to increase the iron content of meat and meat products. Various techniques such as encapsulation, chelation, and microencapsulation have been explored to minimize the negative impact on the sensory qualities of the final product (Câmara et al. 2020; Gao et al. 2020; Lee et al. 2021).

**Fig. 4:**
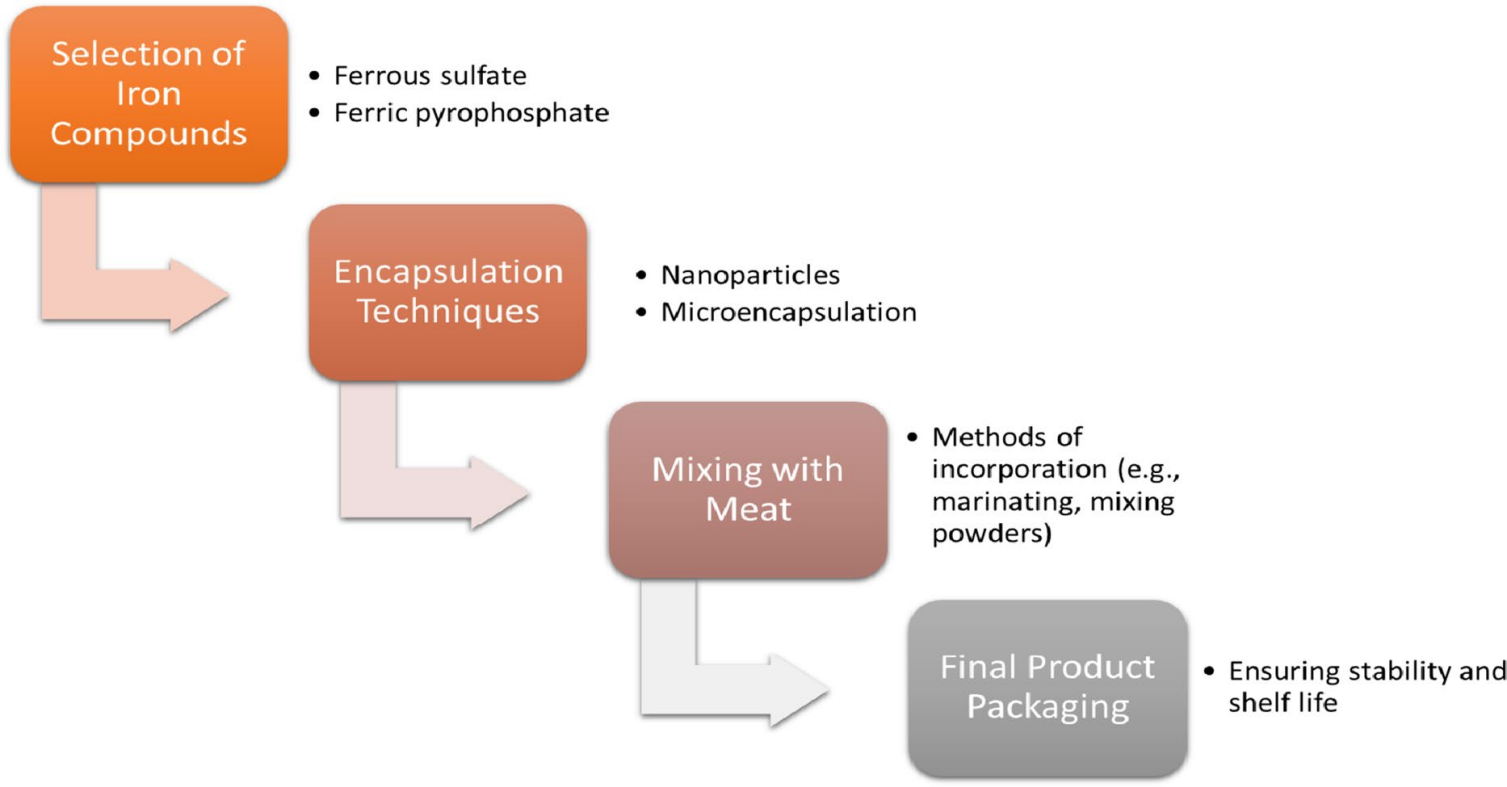
Process of iron fortification in meat products

The fortification of iron in meat and meat products has predominantly been carried out through three techniques: (i) incorporation of iron-fortified ingredients using electrolytic iron, iron amino acid chelates, iron polymaltose, iron hydrolysate, and encapsulated carbonyl iron, which are supplied in the form of powders, paste, and in a microencapsulated form; (ii) using premeasured sachets/pouches used to mix meat products during preparation, and (iii) marinating meat to enhance the absorbability and retention of endogenous nonheme iron (Shubham et al. 2020; Hurrell 2021; Kumari and Chauhan 2022; Kaur et al. 2022) (Fig. 4).

### Encapsulation of iron compounds

The potential use of nanotechnology in food research is currently a focus of significant interest, and the use of nanoparticles, such as zero-valent iron nanoparticles, for the delivery of metal-containing foods via encapsulation or binding to biopolymers is a topic of both current and recent studies (Góral et al. 2023; Noubactep et al. 2023; Białowąs et al. 2024). This has considerable potential for the development of more innovative delivery strategies for iron-containing meat and meat-fortified products. This exciting study provides the first pilot data on the effect of iron-based nanoparticles on *L. monocytogenes* in a meat product model (mini-salami). Food-grade gelatin was used to encapsulate iron nanoparticles and was tested for possible antimicrobial effects using the same model. The nanoparticles did not affect the growth or survival of *L. monocytogenes*, and the quality of mini-salami remained unchanged (Ligaj et al. 2020; Xiang et al. 2021; Monirul Hasan Tipu et al. 2022).

Encapsulation is used to protect chemically reactive or sensitive compounds in a coating that isolates the core material from the surrounding environment and prevents the release of the locked-up material until some form of opening either develops in or completely destroys the encapsulating material. The value of iron encapsulation is that core materials with high reactivity, solubility, and/or bioavailability can be protected until they are in the environment or system for which they are intended. Encapsulation also allows for the opening of the coating that was developed to protect the core material, and the process makes a difference in a controlled manner such that it is timed for in vivo release in the desired location or system. The development of encapsulation-based strategies for iron tailored to the needs of meat-product-based delivery systems has proven challenging and is still in its early phases (Shubham et al. 2020; Kazemi-Taskooh and Varidi 2021; Hu et al. 2022; Zhang et al. 2022).

### Coating technologies

**Fig. 5:**
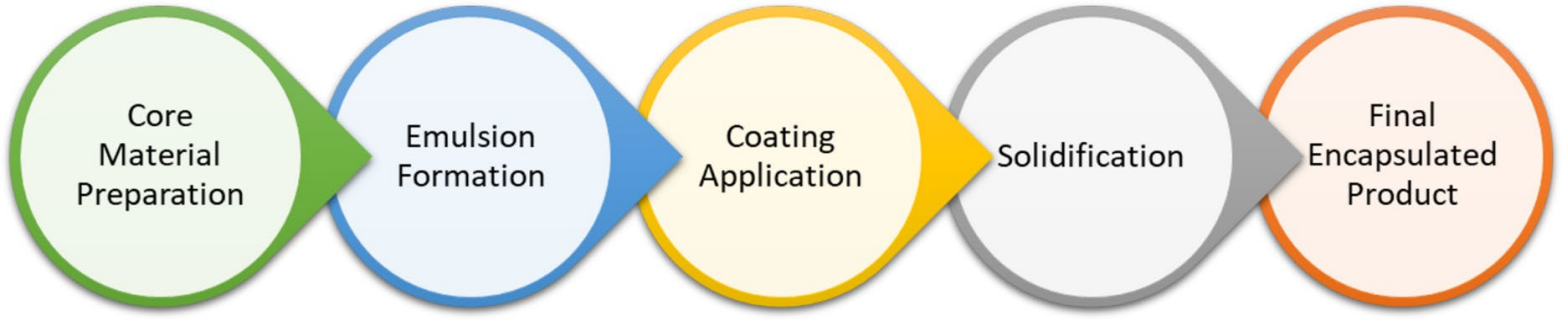
Schematic of coating and microencapsulation process

Numerous complex changes could potentially occur in meat products during the storage of their structural units. During these processes, the quality, nutritional value, and acceptability of meat products fortified with microencapsulated l(+) lactic and l(+) ascorbic acids can be changed by microbial, chemical, enzymatic, photo-oxidative, and oxidative spoilage. A promising approach described in the literature to retard these deteriorative processes and prolong shelf life, thus ensuring the microbial safety, functionality, and nutritional value of a specific food system, is the encapsulation of bioactive ingredients for the purpose of storage, protection, and targeted delivery in fortifying foods. The techniques of encapsulation (using different flavors, processing methods, dosages, encapsulation yields, sourcing and use of wall forming/preventing, maltodextrin and dextrose, and possibly fructose) have verified their sustainability during microencapsulation and are preferred. The application of encapsulated complex compositions (containing intensification of thermomechanical procedures and selection of antimicrobial agents) requires formulations to guarantee the stability and release of bioactive components from the core based on the expected conditions of the food matrix. The choice of encapsulation process and surface-active principles of inclusions suggested with coating technologies and ingredients are elucidated and substantiated. Obtained exclusively functionalized high bioavailability product fortified with amines, iodines, taurines, and other bioactive unprotected components (Hosseini and Jafari 2020; Marcillo-Parra et al. 2021; Domínguez et al. 2021; Munteanu and Vasile 2021; Zhang et al. 2022; Ojeda-Piedra et al. 2022) (Fig. 5).

## Regulatory guidelines and requirements

Work carried out by the Codex Committee on Food Labeling (CCFL) on setting criteria to avoid consumer deception led to the adoption of guidelines on vitamin and mineral food supplements. The Codex guidelines aim to provide advice on the maximum amounts of vitamins and minerals that make them suitable to be marketed as food supplements, considering nutrient reference values. Because these guidelines often include a range of temperature and time combinations, manufacturers can choose a heat process that will fit their production process needs, which falls within the scope of authorization. The table on heat treatments allowed for unprocessed meat was added to the Annex of Nutrient Sources for the same reasons that led EFSA to propose time–temperature combinations to be enclosed in the Annex. The introduction of the table allows a safe and easy approach to the authorization of the best currently known technologies, ensuring respect for otherwise unauthorized vitamins or minerals within the general regulatory framework for the EU. It could flavor frozen meals and could also be used as a source of iron. No heat treatment is implied although when utilizing the commercial cooking process, the pH of the complete product shall not be above 5.6 (O’Neill et al. 2020; Allen et al. 2020; Konings et al. 2021; EFSA 2022; Woźniak et al. 2022; Almeida et al. 2022).

As a food supplement, iron usage in meat products must be regulated by guidelines and legislation. Although there is no World Trade Organization (WTO) reference under the Agreement on the Application of Sanitary and Phytosanitary Measures (SPS), and not part of the Agreement on Technical Barriers to Trade (TBT), several international organizations such as the Codex Alimentarius are responsible for developing food standards, guidelines, and related texts. Codex standards for fortification are not mandatory unless a country decides to follow them, although they are often used as guidance documents. Internal food safety regulations and control of food in the European Union are delegated to the European Food Safety Authority (EFSA). Therefore, the EFSA is responsible for issuing opinions on the safety and efficacy of iron substances in unprocessed meat. Health claims, as part of marketing, are regulated by the European Commission and often follow the EFSA opinion. Once the EFSA issues a scientific opinion, the European Commission acts accordingly and decides whether to enable its use. (Turck and Bohn. 2021; EFSA 2022; Kaur et al. 2022; Fairweather-Tait 2023; Günther 2023; Schöne et al. 2023; Anjum et al. 2024).

## Analytical methods for iron determination in fortified meat products

Iron determination in fortified meat products can be achieved through various analytical methods, each with its own advantages and limitations. High-Performance Liquid Chromatography (HPLC) has been shown to be a rapid and reliable method for simultaneous determination of B vitamins, including iron-containing vitamin B12, in fortified meat products. This method ensures low detection limits, good sensitivity, and resolution (Riccio et al. 2006). For direct iron content analysis, both wet ash and dry ash digestion methods have been employed. The dry ash method has been found to yield approximately double the iron values compared to the wet ash method, due to more complete volatilization of the organic matrix (Windham and Field 2000).

**Fig. 6:**
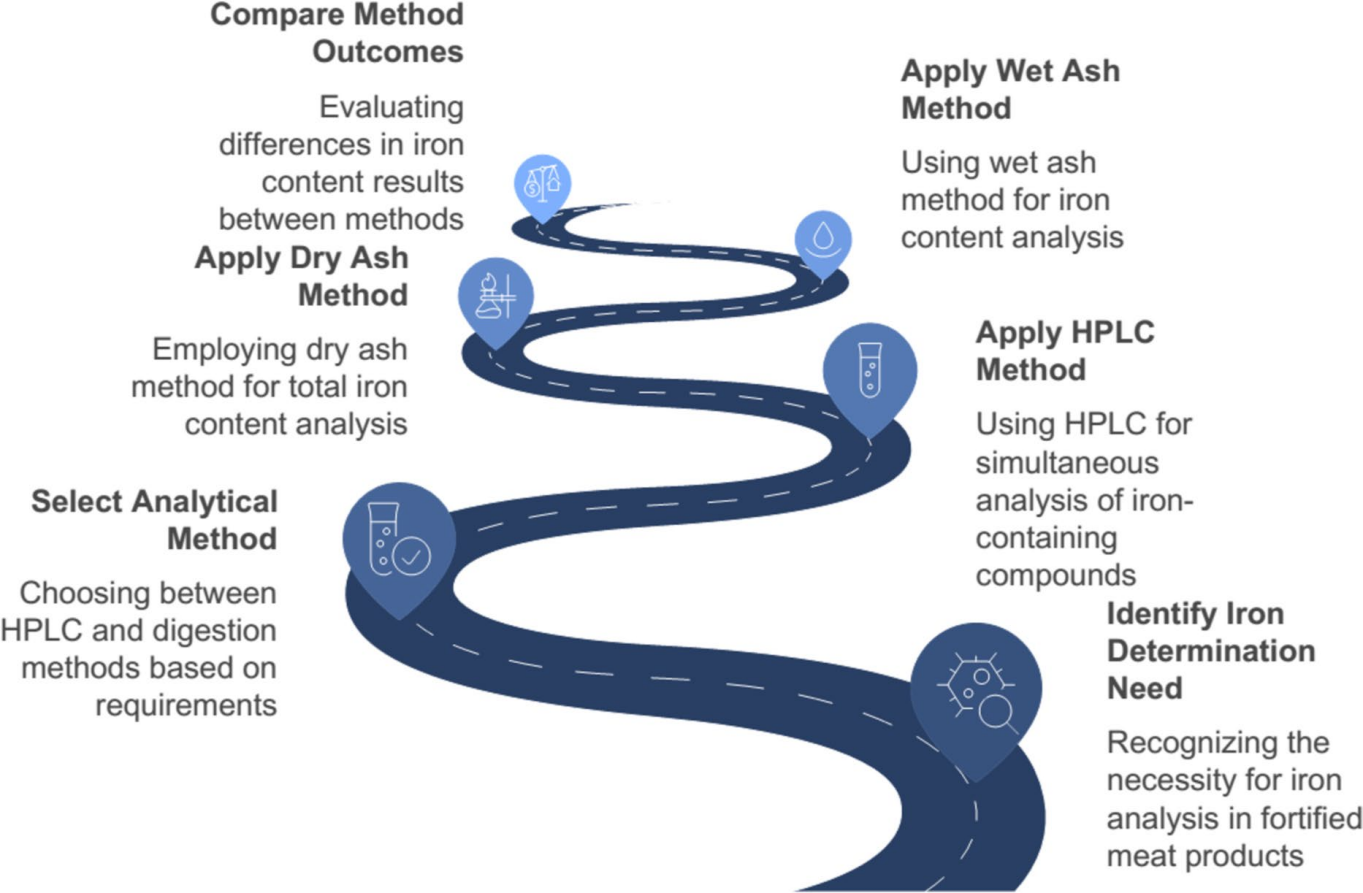
Analytical methods for iron determination

Interestingly, the choice of analytical method can significantly impact the results and subsequent interpretations. For instance, the performance standards developed for added iron in advanced meat recovery (AMR) systems differed substantially between wet and dry ash methods (1.8 mg vs 3.2 mg added iron per 100 g, respectively) (Windham and Field 2000). This highlights the importance of method standardization in regulatory contexts. While HPLC is useful for analyzing iron-containing compounds like vitamin B12, direct iron content analysis typically involves digestion methods. The dry ash method appears to be more accurate for total iron determination. However, the choice of method should be carefully considered based on the specific research or regulatory requirements, as it can significantly influence the results and their interpretation (Fig. 6).

There are literatures that provides a review emphasizing different iron molecules and their interactions in the meat matrix and the primarily used methods of iron determination together with their current limitations for application in food industry for quality and health processing (Reeves 2020; Suehiro et al. 2020; Chakka et al. 2021; Baumgartner et al. 2022; Lee et al. 2024; Xue et al. 2024).

The iron content of a sample can be determined from the total iron content, solubilized iron, or extracted iron content. The total iron content of meat products can be determined by the dry ashing method, in which the sample is subjected to high temperatures to allow organic matter to be completely burned, and the ash can then be dissolved in concentrated acid. However, the content of soluble iron species would be helpful in evaluating the bioavailability of iron. In vitro methods seem to work with mineral extracts and can be used for spectrophotometric or colorimetric (e.g., atomic absorption spectrometry, inductively coupled plasma optical emission spectrometry, or flame emission photometry) detection. The selection of the analytical method depends on the iron form and the characteristics of the fortified products (Soren and Biswas 2020; Schöne et al. 2023; Bou et al. 2024) (Table 1).

**Table 1:** Outlining various analytical methods for iron determination in fortified meat products, including the advantages and limitations for each method

Method	Advantages	Limitations
Atomic absorption spectroscopy (AAS)	– High sensitivity and precision – Capable of detecting trace amounts of iron – Relatively simple sample preparation	– Requires expensive equipment – Cannot differentiate between different iron species (e.g., heme vs non-heme iron)
Inductively coupled plasma optical emission spectrometry (ICP-OES)	– High throughput and sensitivity – Multi-element analysis possible – Suitable for large-scale testing	– High operational cost – Requires specialized equipment and trained personnel
Inductively coupled plasma mass spectrometry (ICP-MS)	– Ultra-high sensitivity – Capable of detecting low levels of iron – Multi-element detection	– Very expensive equipment – Complex sample preparation – Prone to contamination errors
Spectrophotometric methods (e.g., Ferrozine Assay)	– Cost-effective – Simple and widely available instrumentation – Suitable for routine analysis	– Less sensitive compared to AAS and ICP techniques – Can be affected by sample matrix interference – Requires careful calibration
X-ray fluorescence (XRF)	– Non-destructive analysis – Fast and minimal sample preparation – Capable of analyzing solid samples directly	– Limited sensitivity compared to ICP and AAS – Less accurate for low iron concentrations
Titrimetric methods (e.g., redox titration)	– Simple and inexpensive – No need for sophisticated equipment – Suitable for large sample sizes	– Less sensitive and accurate compared to instrumental methods – Requires manual handling, increasing human error potential
Colorimetric methods (e.g., thiocyanate method)**	– Relatively simple and quick – Inexpensive reagents and equipment	– Interference from other metals can affect accuracy – Lower sensitivity and precision compared to instrumental methods
Electrochemical methods (e.g., potentiometry, voltammetry)**	– High sensitivity – Can differentiate between different oxidation states of iron (Fe^2^⁺ vs Fe^3^⁺)	– Requires specific electrodes and careful calibration – Susceptible to interference from other ions in the sample

## Bioavailability of iron in fortified meat products

The use of iron as a food additive to fortify food products is an effective method to increase the level of iron in the human body. This measure is particularly important for developing countries. Iron is usually added to products such as milk, milk-based foods, soft drinks, juices, and meat. Enterostomal cells, which are located on the apical part of the villi of the small intestine, are the main recipients of assimilated iron in the human body. Iron has several functions, such as myoglobin and the mitochondrial respiratory chain of mitochondrial muscles; however, most of the iron obtained from food is used differently. Women of childbearing age, particularly pregnant women, have the highest need for iron because the rapidly growing fetus consumes the majority of the maternal iron resources. Increased consumption of cow's milk can also be a concern because it consumed in large quantities can cause lower portal iron absorption, which is most noticeable in infants and children (Fairweather-Tait 2023; R. F. Hurrell 2021; Liberal et al. 2020; Perera et al. 2023; Sun and Weaver 2021) (Table 2).

**Table 2:** Types of iron compounds used in fortification

Iron compound	Bioavailability	Advantages	Disadvantages
Ferrous sulfate	High	Cost-effective, high absorption rate	Can cause taste alterations
Ferric pyrophosphate	Moderate	Stable, minimal taste impact	Lower absorption rate compared to ferrous sulfate
Iron amino acid chelates	High	High bioavailability, less taste impact	More expensive
Encapsulated iron	Variable	Controlled release, protection from oxidation	Complex production process

Iron is an essential nutrient, and its compounds are widely used to fortify various food products. This study reviews research published over the past 10 years on the use of iron compounds in meat products, the effects of these compounds on the quality of meat products, and the bioavailability of iron in these products. The review is not exhaustive, but serves as an example of the possibility of applying specific iron compounds to fortify meat products with iron. The stability of iron additives in foods and the interactions between iron and other food components that affect iron bioavailability were also discussed. Special attention has been paid to the legal aspects and general safety of using iron supplements in meat products. The growing public interest in healthy food and the resulting need to develop new methods to fortify animal products with elements that promote health have been discussed (Shubham et al. 2020; Hurrell 2021; Kumari and Chauhan 2022).

In conclusion, fortified meat products can be an effective strategy for improving iron status, especially when combined with other fortified foods or enhancers like vitamin A. Therefore, when designing iron-fortified meat products, it is essential to consider factors such as the type of iron compound, the presence of enhancers, and the target population's health status to maximize iron bioavailability and effectiveness.

## Sensory aspects of iron-fortified meat products

The choice of iron compound is crucial for maintaining sensory acceptability. Micronized ferric pyrophosphate was found to produce extruded rice grains with excellent sensory characteristics and potential high bioavailability (Moretti et al. 2005). NaFeEDTA was preferred as the most suitable iron fortificant for dehulled lentils across five sensory attributes (Podder et al. 2018).

Another way to change the color of iron to a more attractive color is to use packaging such as modified atmosphere packaging. Instead of iron fortification, the development of mainly vegetarian and other iron-enriched meat alternative products containing whole-grain cereals, legumes, and seeds was observed. Consideration of the iron loss or bioavailability for which iron fortification is effective in meat and meat products is still necessary. Critical examination of the nutrient composition of meat alternatives based on ferritin, iron, myoglobin, or hemoglobin was neglected. Only myoglobin is used as a meat model, and meat models for hematopoietic, hematological, and related functions have not been evaluated for meat alternatives or vegan diets. Iron fortification produces aroma and taste effects, such as lipid oxidation and iron. The use of natural antioxidants and iron is recommended in the meat industry. Therefore, this study will have a huge impact on further research and advice on meat fortification (Shubham et al. 2020; Hurrell 2021; Kumari and Chauhan 2022; Mayer Labba et al. 2022; Fairweather-Tait 2023) (Table 3).

**Table 3:** Sensory and nutritional impact of iron fortification

Fortification technique	Sensory impact	Nutritional impact	Overall consumer acceptance
Encapsulation	Minimal taste alteration	High bioavailability of iron	High
Direct mixing	Possible taste and color changes	Moderate to high depending on iron compound	Moderate to high
Marination	Enhanced flavor and iron absorption	High bioavailability	High

**Fig. 7:**
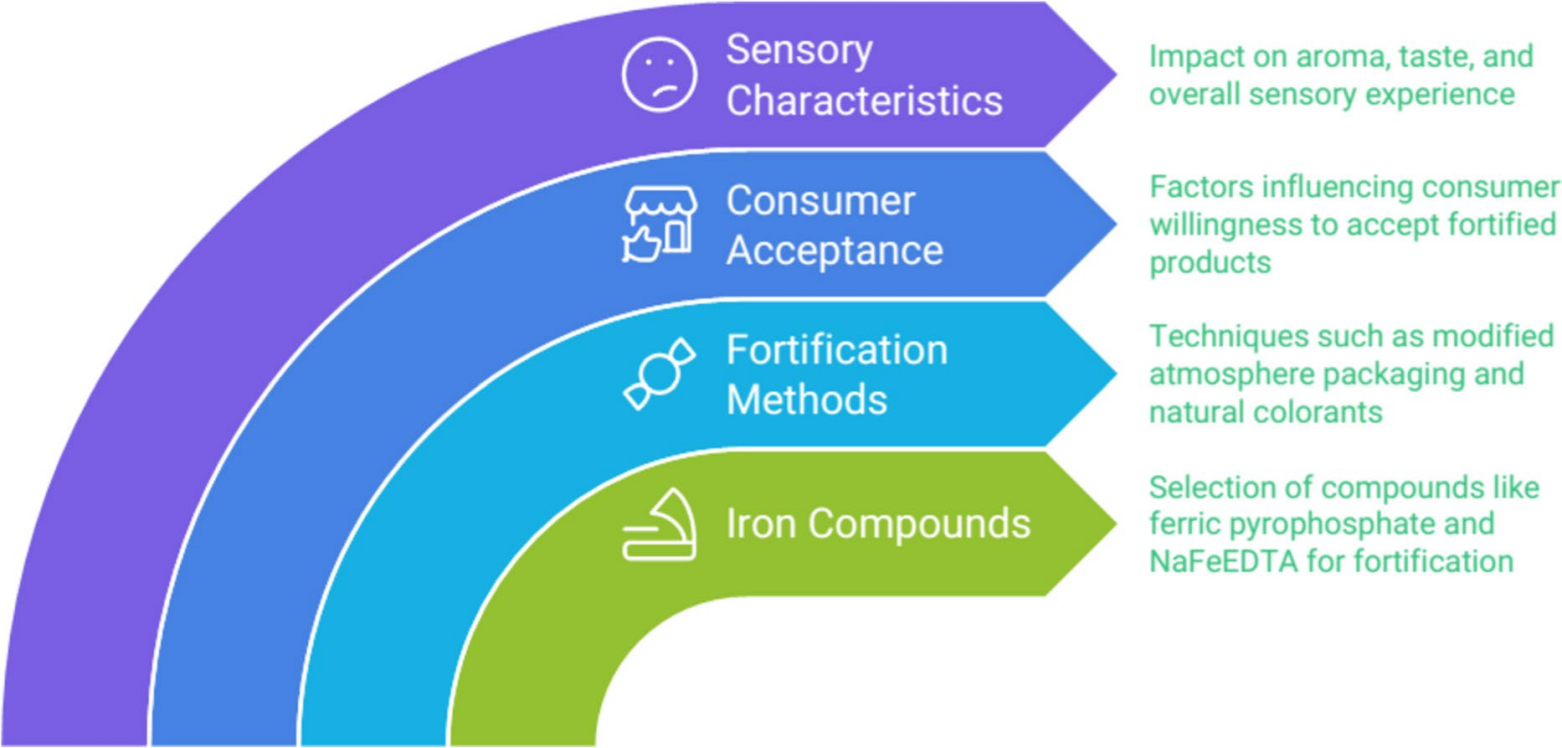
Enhancing sensory acceptability in iron fortification

The attitude of the consumer is one of the most important issues that researchers encounter during studies on iron, sodium, and other critical minerals in various foods. In populations at a high risk of iron deficiency, most consumers accept fortified meat. Replacement of metmyoglobin, which tends to discolor iron-fortified meat, with natural colorants such as anthocyanins was very effective. Fermentation with *Rhizomucor miehei*, which produces white discoloration in pork sausages, has been proven to be a successful method. The use of a lower level of nitrogen, resulting in a desirable end color, has led to consumer acceptance (Rizwan et al. 2021; Fairweather-Tait 2023; Welk et al. 2023; Embling et al. 2024) (Fig. 7).

In conclusion, while iron fortification can affect sensory properties, careful selection of iron compounds and fortification methods can help minimize negative impacts on product acceptability. However, specific information on iron-fortified meat products is not available in the provided context.

## Consumer acceptance and perception

Consumer acceptance and perception of iron-fortified meat products are influenced by various factors, including nutritional knowledge, sensory attributes, and product information. Research indicates that consumer perception of iron-fortified foods is associated with nutrition knowledge. A study found that a one-point increase in overall nutrition knowledge score was linked to a 4.3% higher likelihood of believing iron-fortified foods have a positive role in diet (Pounis et al. 2011). Moreover, increased iron nutrition knowledge was associated with a 20% higher likelihood of consuming iron-fortified foods more frequently (Pounis et al. 2011).

Sensory evaluations have shown promising results for iron-fortified products. In a study involving toddlers and their mothers, porridge made from maize fortified with ferrous bisglycinate (BIS) was well-accepted, with no significant differences in overall liking compared to unfortified maize (Bovell-Benjamin et al. 1999). Similarly, iron-fortified Ultra Rice (UR) showed no significant sensory differences from conventional rice, with mean acceptance scores ranging from "liked" to "really liked" (Beinner et al. 2010).

The main factors that influence product acceptance are the intrinsic attributes of the foods, which are well known and well resolved and must be maintained or improved, along with compliance with personality characteristics, cultural factors, gender, age, knowledge level, and socioeconomic condition of the consumers. In the population, especially in females and children, there may be more significant iron deficits; therefore, it is essential to encourage the intake of iron-rich products in these groups to help improve the physiological and psychological aspects of these individuals. Satisfactory results increase the chance of a better nutritional status but only maintain the adequacy of the intake of iron-rich foods and consider all the sensory properties, as evaluated by the humorous analysis carried out economically, which will enable the production and marketing of a food that satisfies the consumer (Muriuki et al. 2020; Andriastuti et al. 2020; Zheng et al. 2020; Benson et al. 2021; Turawa et al. 2021).

The concept of acceptance is related to the evaluation of the quality of the product in all its aspects, including color, taste, and aroma, through sensory analysis techniques and not to the acceptance that is associated with an individual's attitude toward the product. The word liking refers to liking what is known to be of poor quality at objectively identified quality levels, for example, high-fat foods, and has properties that have to be independent. However, there is evidence that people do not normally separate the subjective concept of acceptance from that of sensory liking. (Podder et al. 2020; Saldaña et al. 2021; Sparvoli et al. 2021; Ngume et al. 2022).

**Fig. 8:**
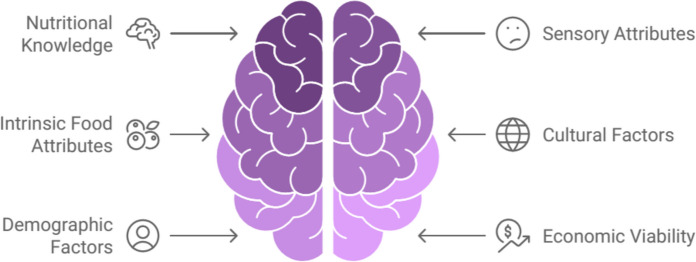
Factors influencing consumer acceptance

The fortification of foods with iron has positive economic viability but can cause changes in the sensory and technological characteristics of foods. The consumer is usually the main evaluator of foods; therefore, he has determined the sensory properties of products and has the leading role in the purchase; thus, it is clear the necessity for good consumer acceptance of the product for the fortification to be successful (Cappellini et al. 2019; Freeland-Graves et al. 2020; Al-Naseem et al. 2021; Kumar et al. 2022) (Fig. 8).

In conclusion, consumer acceptance of iron-fortified meat products appears to be influenced by nutritional knowledge and sensory attributes. Educating consumers about the benefits of iron fortification and ensuring that the sensory qualities of fortified products match or exceed those of conventional products may be key to increasing acceptance. However, it's important to note that the available research focuses more on iron-fortified grains rather than meat products specifically, indicating a need for further studies in this area.

## Health benefits and risks of iron-fortified meat products

Iron fortification of foods, including meat products, is a cost-effective strategy to prevent iron deficiency anemia, a global public health problem affecting 40% of children and 30–37% of women worldwide (Blanco-Rojo and Vaquero 2019; Perera et al. 2023). Iron-fortified foods have shown good potential to improve population iron status, with efficacy studies reporting positive impacts on hemoglobin levels and reduced risks of anemia and iron deficiency (McAfee et al. 2010).

In conclusion, while iron fortification of meat products can be an effective strategy to combat iron deficiency, careful consideration must be given to the level of fortification and the target population. Moderate consumption of lean red meat as part of a balanced diet is unlikely to increase risks of cardiovascular disease or colon cancer and may positively impact nutrient intakes and fatty acid profiles (McAfee et al. 2010). Future research should focus on developing iron-rich ingredients with high bioavailability, better stability, and lower cost, as well as newer processing technologies for more effective fortification (Man et al. 2021).

There are safer and more effective alternatives to the prophylactic fortification of all foods, such as supplementation for those identified as having an inadequate status. This prevalence can also be reduced using other means. Iron consumption is positively associated with body iron status; therefore, the prevalence of ID can be reduced by increasing the iron intake. Although there is evidence that a substantial proportion of women achieve their requirements through animal sources, the specific requirements for haem iron absorbance are still unclear. Outputs from the Cochrane review of the effects of iron supplementation, fortification, and dietary diversification on hemoglobin, ferritin, and soluble transferrin receptors were included. This review concluded that dietary diversification was more beneficial as an intervention; iron fortification was mostly beneficial in regions where a high prevalence of infections rendered iron supplements unfavorable, and iron supplementation was likely to have a higher impact than dietary diversification or iron fortification (Waller et al. 2020; Pasricha et al. 2020; Man et al. 2021; Hurrell 2022).

This review outlines the problem of iron deficiency and iron deficiency anemia and presents evidence that meat is an important part of the solution. Because of its high bioavailability, haem iron from meat has a disproportionately large effect on iron status compared to its contribution to the diet. Projects aimed at improving the nutritional status often focus heavily on improving the nutritional quality of non-meat products, demonstrating a vegetarian or vegan bias. This has led to initiatives such as fortifying cereals and bread with iron. Meat remains the second largest source of iron in the global diet but is often overlooked, while increasing meat consumption is part of the remedy for undernutrition (Abioye and Fawzi 2020; Callister et al. 2020; Chouraqui 2022; Consalez et al. 2022; Brittenham et al. 2023; Von Holle et al. 2023).

## Case studies and success stories

Iron deficiency anemia is an increasingly recognized global problem, and its prevalence results in substantial human morbidity. The provision of iron, either alone when anemia prevails or alternatively through iron-fortified food, is significantly important for millions of people worldwide, particularly for women of childbearing age, pregnant women, children, and infants. Fortifying foods with iron can be cost-effective for preventing iron deficiency. However, some iron compounds that are effective in preventing or curing iron-deficiency anemia through rectified fortification are unsuitable and have unacceptable adverse sensory attributes. Meat and meat products may be alternatives. Animals that have been fed iron-enriched feed, as well as raw meat steaks, juices, and sauces, all contain large amounts of bioavailable heme iron. In many countries, middle- and upper-income groups regularly eat red meat, particularly beef, lamb, and pork, but these products are not accessible or costly, particularly for deprived populations. Meat or meat products in different forms (e.g., ready-to-eat, non-perishable, fast-food, and semi-durable) are widely consumed. In order of preference, pork, poultry, and beef products are consumers' preferred ready-to-eat meat products. In an attempt to control iron deficiency anemia, meat and meat products offer the finest chance in a large number of categories. Regrettably, a negligible number of reports have been published and focused on iron-enriched meat and meat product development programs (Cappellini et al. 2019; Owaidah et al. 2020; Bathla and Arora 2021; Natekar et al. 2022; Kumari and Chauhan 2022; Munro 2023).

Iron deficiency anemia (IDA) is recognized as one of the most widespread malnutrition problems worldwide. The iron fortification of staple food products aims to prevent and help control this nutritional deficiency. Food fortification has undergone exceptional growth in recent years. Nevertheless, only a small number of food products are fortified with iron. The success of any fortification program is directly related to the acceptability of the product and its availability to the public. Meat and meat products are universally consumed, possess high nutritional value for humans, and have the potential to be utilized as vehicles for iron fortification. In this context, this review presents an alternative for the control of iron deficiency anemia using iron-fortified meat and meat products (Kumari and Chauhan 2022; Mayer Labba et al. 2022; Fairweather-Tait 2023; Perera et al. 2023).

## Future trends and innovations in iron fortification

Iron fortification remains a crucial strategy to combat iron deficiency worldwide, with ongoing research focusing on improving efficacy and overcoming existing challenges. Several innovative approaches are emerging as future trends in iron fortification:

Biofortification through genetic engineering is showing promise as a sustainable solution. Researchers have successfully increased iron content in rice endosperm by introducing a ferritin gene from Phaseolus vulgaris, while also improving bioavailability by incorporating a thermo-tolerant phytase and overexpressing cysteine-rich proteins (Lucca et al. 2002). This approach has the potential to substantially improve iron nutrition in populations where deficiency is widespread.

Nanotechnology is being explored for developing new iron fortificants, innovative complexes, and coatings to enhance bioavailability and reduce sensory changes in fortified foods (Blanco-Rojo and Vaquero 2019). These advancements may help overcome the limitations of traditional iron compounds used in fortification.

Precision nutrition is gaining traction, aiming to identify vulnerable groups based on genotype, dietary habits, physical activity, and metagenomic profiles (Blanco-Rojo and Vaquero 2019). This approach could lead to more targeted and effective iron fortification strategies tailored to specific population segments.

Interestingly, research indicates that iron fortification alone may have limited impact on iron status in diets with low bioavailability. Combining fortification with dietary modifications shows more promising results, with potential increases in iron stores up to 70% greater than either strategy alone (Hoppe et al. 2007). This suggests that future iron fortification programs may need to adopt a more holistic approach, integrating dietary education and modification alongside fortification efforts.

**Fig. 9:**
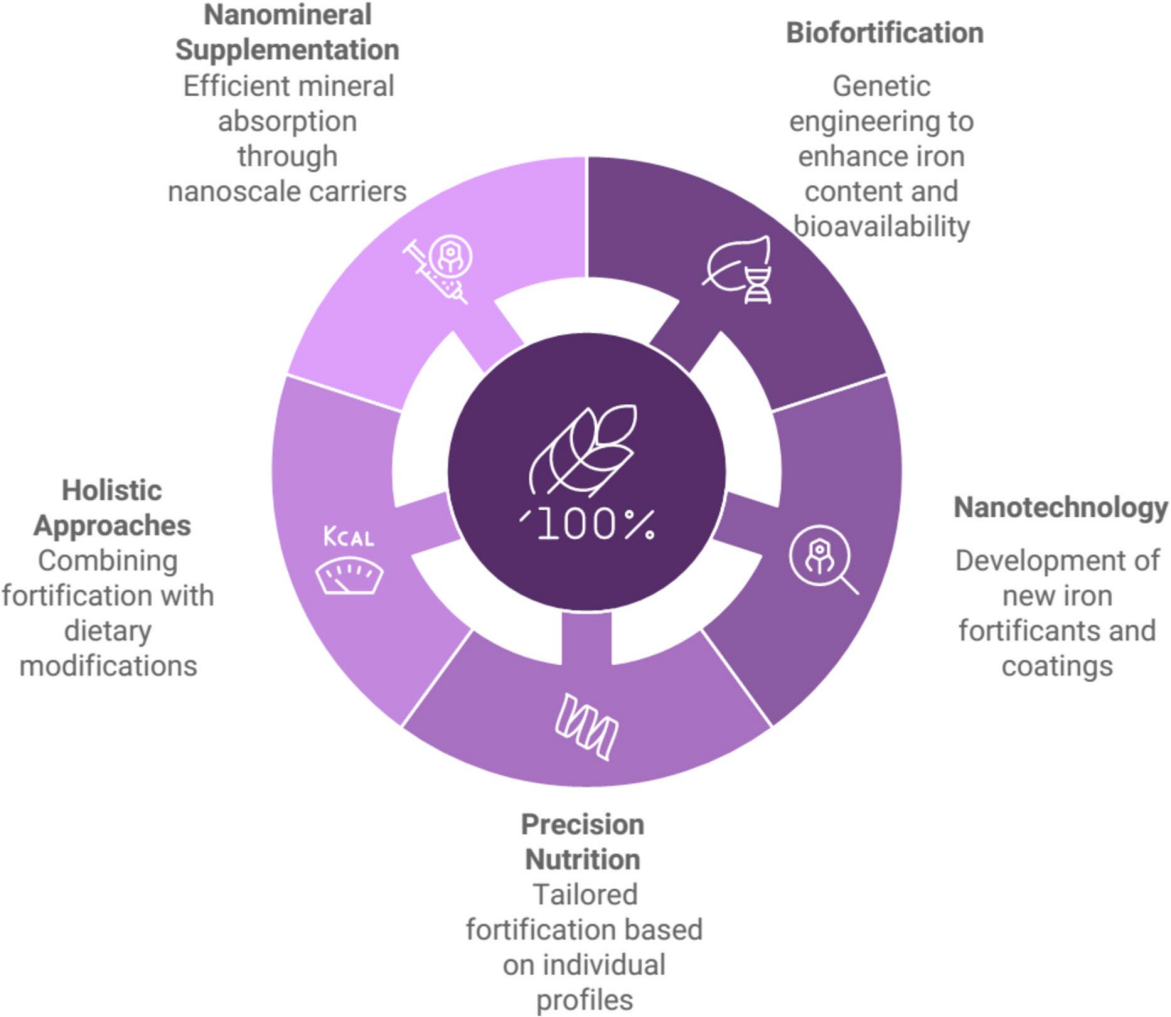
Future trends in iron fortification

Nanomineral supplementation is a strategy to alleviate mineral deficiencies because minerals can be absorbed very efficiently in the human body at the nanoscale. The metal requirements in humans can be satisfied by administering sufficient metal quantities within the range of 1–100 nm. The only well-established clinical anemia therapy is treatment with drug-loaded liposome-based formulations to combat iron-deficiency anemia. Further studies are needed to confirm and understand the therapeutic value of nanoscale carriers. Moreover, the potential toxicity of these nanoscale carriers should be carefully addressed before their translation into humans. The percentage of anemia in the population will be in control, and this will, in turn, protect individuals from oxidative stress and thus save many lives due to food fortification with nanoparticles and nanocomplexes (Patra and Lalhriatpuii 2019; Singh and Onuegbu 2020; Sarathi Swain et al. 2021; Abdelnour et al. 2021; Bhagat and Singh 2022) (Fig. 9).

In the coming years, food products will be developed using alternative and sophisticated technologies that deliver the maximum benefits of added nutrients without altering the sensory properties and inducing the fear of products lacking naturalness. Over time, the biggest change would be food fortification with ingredients that offer greater bioavailability, are better tolerated by the consumer, and can be used at lower doses or have no technological limitations. Nanoscience offers unique tools and techniques at the nanometer scale for the production of a new generation of well-defined nanosized iron supplements and fortifiers (Nile et al. 2020; Anilakumar 2021; Lamri et al. 2021; Ulloa-Saavedra et al. 2022).

## Conclusion and recommendations

Iron is an essential mineral; however, dietary iron deficiency is a common nutritional problem worldwide. Anemia affects approximately 20–25% of the world's population and 30–50% of pregnant women. Iron is especially important for (pre-) school children, as it influences cognitive development. Meat and meat products universally contain substantial amounts of iron; however, red meat consumption should be reduced for health reasons. In some countries, this results in a substantially decreased iron intake from meat. Iron can be supplied through fortified foods. Meat normally does not require fortification, but food-grade iron compounds, such as ferric pyrophosphate, can be added to meat products, such as meatballs, up to maximum levels of approximately 500 mg iron per kg. The meatball recipe should be adjusted to minimize the undesirable mechanical and optical impairments induced by the ingredients added for fortification. Ferric pyrophosphate has excellent bioavailability, and its beneficial changes in meat product formulas can be explained by color and mineral analyses as well as theoretical considerations regarding the behavior of colored meat products. Optimization approaches for the sensory quality of fortified meatballs and new preservation options, such as controlled temperature abuse with further increased iron uptake by meatball consumers or natural clostridial inactivation, are discussed. The future of iron fortification lies in multifaceted approaches combining advanced technologies, precision nutrition, and dietary modifications. As research progresses, these innovations are expected to enhance the efficacy and sustainability of iron fortification programs worldwide.

## Data Availability

No datasets were generated or analysed during the current study.
